# 

**Published:** 1878-03

**Authors:** 


					﻿MARCH, 1878.
TWENTY-FIFTH YEAR—No. 291.
HALL’S
Journal of Health.
PUBLISHED AT BIBLE HOUSE,
THIRD AVENUE, CORNER OF NINTH STREET,
NEW YORK.
E. H. GIBBS, M.D., Editor.
AGENTS FOR THE TRADE I
AMERICAN NEWS COMPANY, 121 NASSAU STREET.
NEW YORK NEWS COMPANY, 18 BEEKMAN STREET.
Terms, $1.50 a Year, or $1.00 for Eight Months. Postage paid.
SINGLE NUMBER, 15 CENTS.
				

